# Recurrent spinal primitive neuroectodermal tumor with brain and bone metastases: A case report

**DOI:** 10.1097/MD.0000000000008658

**Published:** 2017-11-17

**Authors:** Frank Chen, Shyh-Shin Chiou, Sheng-Fung Lin, Ann-Shung Lieu, Yi-Ting Chen, Chih-Jen Huang

**Affiliations:** a Department of Radiation Oncology; b Department of Pediatrics; c Department of Internal Medicine; d Department of Surgery; e Department of Pathology, Kaohsiung Medical University Hospital, Kaohsiung Medical University; f Faculty of Medicine, College of Medicine, Kaohsiung Medical University, Kaohsiung, Taiwan.

**Keywords:** metastases, PNET, radiotherapy, recurrence, spinal primitive neuroectodermal tumor, tomotherapy

## Abstract

**Rationale:**

Primary spinal primitive neuroectodermal tumor (PNET) is relatively rare in all age groups, and the prognosis in most cases of spinal PNETs appears to be poor, with a median patient survival of 1 to 2 years. We present a case with recurrent spinal PNET with brain and bone metastases that was successfully treated by multimodality treatment.

**Patient concerns:**

A 14-year-old teenage girl had suffered from progressive left upper back pain with bilateral lower legs weakness and numbness for 1 year. After treatment, left neck mass was noted 3 years later.

**Diagnoses:**

Initially, magnetic resonance imaging (MRI) showed neurogenic tumor involving intradural extramedullary space of T5-T10. Pathology report showed PNET (World Health Organization grade IV) featuring lobules of neoplastic cells with round regular nuclei, high nucleus-to-cytoplasm ratio, and fibrillary cytoplasm. At the time of tumor recurrence, chest MRI then showed recurrent tumor at T2-T3 level of the epidural space with right neural foramina invasion. Brain MRI showed extensive bilateral calvarial metastases and leptomeningeal metastases in the right frontoparietal regions. Bone scan showed multiple bone metastases.

**Interventions:**

T-spine tumor removal and adjuvant radiotherapy (RT) to T-spine tumor bed were performed in the initial treatment. After clinical tumor recurrence, tumor removal was done again. She then received chemotherapy followed by whole brain irradiation with hippocampal sparing with 35 gray in 20 fractions.

**Outcomes:**

After treatment, follow-up images showed that the disease was under control. There was no neurological sequela. She has survived more than 7 years from diagnosis and more than 4 years from recurrence to date.

**Lessons:**

Multimodality treatments including operation, RT, and chemotherapy should be considered in the initial treatment planning, and salvage chemotherapy was useful in this case.

## 1 Introduction

Primitive neuroectodermal tumor (PNET) is a malignant neoplasm comprising small, undifferentiated neuroectodermal cells. Its common origin sites are the long bones, such as the femur and humerus, and also the pelvic bones. PNET may also occur in the liver, kidneys, and adrenal glands. It belongs to the Ewing sarcoma family, and most commonly arise in adolescents or young adults who are under 35 years of age and has a slight male preponderance.^[1]^ Although PNET is the second most common malignant neoplasm in childhood, the spinal cord, as primary site for PNET, is relatively rare in all age groups.^[2]^ A large series of 430 patients showed that PNETs represent less than 1% of primary spinal tumors,^[3]^ and according to the data from Taiwan Health Promotion Administration, there were only 2 patients with newly diagnosed PNET of nerve system in 2013. With reviewing publications, most reports on PNET focused on supratentorial PNET, whereas PNET affecting spinal location is extremely rare.^[4]^ Primary spinal PNETs are most prevalent in the pediatric and young-adult populations with the median age at the time of diagnosis being 24 years, and are observed more commonly in males than in females with a nearly 2:1 male preponderance. The prognosis in most cases of spinal PNETs appears to be poor with a median patient survival of 1 to 2 years. Approximately one-third of patients will exhibit cerebrospinal dissemination of their tumor.^[5]^

Due to the low incidence of these tumors, there are currently no standard clinical guidelines outlining their management.^[2]^ A reviewed article concluded that recommended treatment for spinal PNET should include decompressing neural elements to prevent further neurological decline, obtaining an adequate tissue sample for pathological examination, and resecting as much tumor as can be safely removed. Evidence of benefit from adjuvant therapy in treating spinal PNETs is not well-established at present. The optimal radiation strategy, the necessity of full neural axis radiation in the setting of localized disease, and dosing are all controversial topics in the treatment of primary spinal PNETs.^[5]^ Craniospinal radiotherapy (RT) and a radiation boost to the primary tumor after surgery might be an option due to the possibility of PNET disseminating along the neuraxis.^[6,7]^ Regimens that combine high-dose chemotherapy and autologous stem cell rescue in addition to surgery and radiation have shown promising results in several reports.^[5]^ The usual agents used include vincristine, ifosfamide, doxorubicin, etoposide (VIDE), methotrexate, cisplatin, and lomustine.^[5,7]^ While choosing adjuvant treatment modalities, treatment-related adverse events should be take into account. RT to the spine and whole neural axis might cause growth delay and cognitive impairment in children, whereas chemotherapy might cause encephalopathy, nausea, bone marrow suppression, peripheral neuropathy, hepatotoxicity, and cardiomyopathy.

Here, we present a case with recurrent spinal PNET with brain and bone metastases that was successfully treated by tumor resection followed by regional adjuvant RT without chemotherapy at first, and received further surgery, chemotherapy, and whole brain RT after local regional and distant metastases.

## 2 Case report

A 14-year-old teenage girl without any underlying disease had suffered from progressive left upper back pain with bilateral lower legs weakness and numbness for 1 year. She was admitted to our hospital in May 2009, where computed tomography (CT) of the chest demonstrated enhanced lesions in the epidural or intradural extramedullary space at the T7-T9, with left extraforaminal extension at the level of the T8 and T9 with compression to the spinal cord, and schwannoma was suspected. Magnetic resonance imaging (MRI) showed neurogenic tumor (eg, schwannoma) involving intradural extramedullary space of T5-T10 with compression of spinal cord, extension, and widening via left T7 and T8 neuroforaminal and nerve roots to the left paraspinal region. T6-T9 tumor removal was performed. Pathology report showed PNET (World Health Organization [WHO] grade IV) featuring lobules of neoplastic cells with round regular nuclei, high nucleus-to-cytoplasm ratio, and fibrillary cytoplasm, while Homer Wright rosettes and calcification were seen. These cells had positive immunostaining for synaptophysin and neuron-specific enolase, and negative immunostaining for glial fibrillary acidic protein. After operation, she received adjuvant RT with 50 gray (Gy) in 25 fractions to T6-T9 spine surgical bed. At that time, her family was concerned about side effects of chemotherapy, so no adjuvant chemotherapy was administered then.

**Figure 1:**
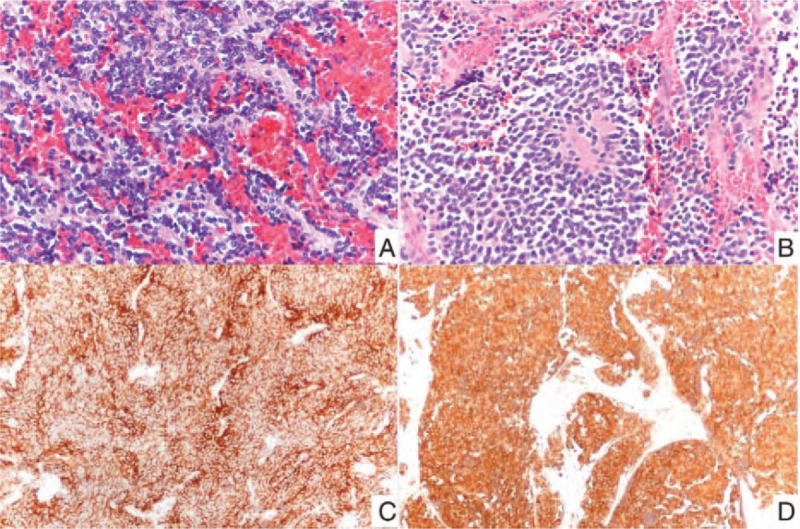
Pathologic images of tumor. (A) T-spine and (B) neck tumor cells featured uniform small blue round nuclei, fine chromatin, high nucleus-to-cytoplasm ratio ,and scant cytoplasm, arranging in rosette pattern. These tumor cells had positive immunostaining for (C) synaptophysin and (D) neuron-specific enolase.

During regular follow-up, however, left neck mass was noted 3 years later. Chest MRI showed recurrent tumor at T2-T3 level of the epidural space with right neural foramina invasion and metastatic lymphadenopathies at left supraclavicular region. The pathology from excision of recurrent tumor revealed PNET with neck lymph node metastasis, as the pathology featured uniform small blue round nuclei, fine chromatin, high nucleus-to-cytoplasm ratio, and scant cytoplasm, arranged in rosette pattern with positive immunostaining for synaptophysin and neuron-specific enolase (Fig. 1). Bone marrow biopsy showed focally positive immunostaining for cluster of differentiation 99. Cerebrospinal fluid aspiration was done for her, and no malignant cell was identified. After surgery, brain MRI showed extensive bilateral calvarial metastases with dural involvement and leptomeningeal metastases in the right frontoparietal regions. Bone scan showed bone metastases from PNET in progress.

**Figure 2:**
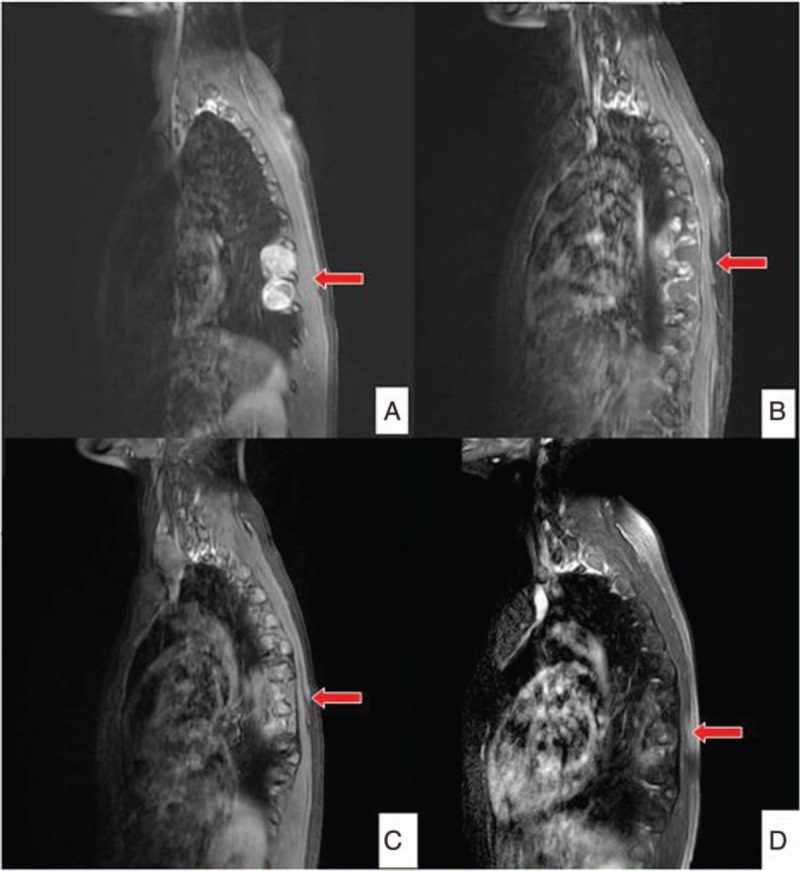
Serial images of T-spine magnetic resonance imaging. (A) T-spine tumor first noted in 2009. (B) After first tumor removal. (C) Recurrence of T-spine tumor. (D) Follow-up image after second operation in 2015 (red arrows indicate the lesions).

She then received 7 cycles of chemotherapy with VIDE, followed by cyclophosphamide with topotecan for 7 cycles. After finishing chemotherapy, brain MRI showed residual bilateral calvarial metastases with dural involvement, whereas spine MRI showed no significant change of postoperative fluid accumulation around the left T7-T9 paraspinal area and suspicious postoperative granulation around left the T7-T8 paraspinal area (Fig. 2).

**Figure 3:**
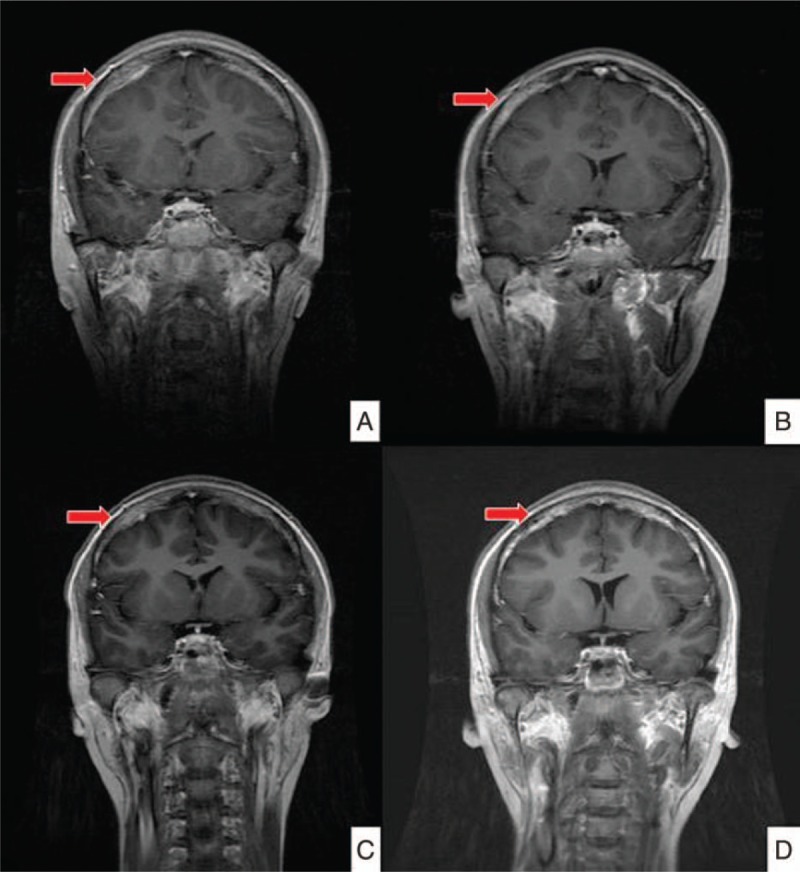
Serial images of brain magnetic resonance imaging. (A) Newly found brain metastasis in 2013. (B) Improvement of brain metastasis after chemotherapy. (C) Residual brain metastasis in 2014. (D) Follow-up image in 2015 after whole brain radiotherapy in 2014 (red arrows indicate the lesions).

**Figure 4:**
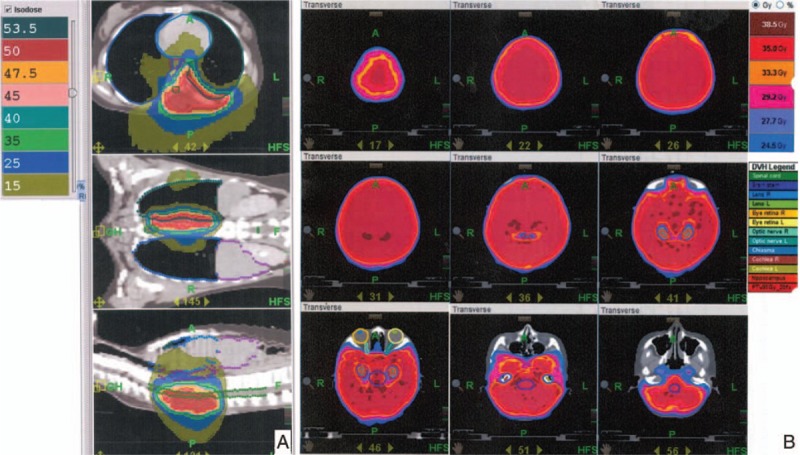
Dose distribution of radiotherapy. (A) Dose distribution of radiotherapy to T-spine surgical bed by Tomotherapy in 2009. (B) Dose distribution of whole brain irradiation with hippocampal sparing by Tomotherapy in 2014.

During follow-up, relapse of brain metastasis was noted. For better local-regional control, whole-brain RT with hippocampal sparing was done with 35 Gy in 20 fractions. After treatment, follow-up images showed disease under control. There was no neurological sequela observed during follow-up at outpatient department. She has survived more than 7 years from diagnosis and more than 4 years from recurrence to date (Figs. 3 and 4).

The patient signed and provided written informed consent for publication of this report and all accompanying images.

## 3 Discussion

In 1918, Stout et al reported that a PNET is an ulnar nerve-derived small round cell tumor with rosette formation that develops in soft tissues in young individuals. Separately, James Ewing reported a rare type of sarcoma in children, adolescents, and young adults that is a highly malignant tumor of the bone and soft tissue, composed of small round cells in 1921. The first small round cell tumor was reported in a pediatric patient by Askin, and these tumors are referred to as Askin tumors. With recent improvements in molecular biology, Ewing sarcoma and PNET were subsequently integrated into a single item in the WHO classification in 2007.^[8]^ Ewing sarcoma was thought to be a malignant tumor originating from marrow mesenchymal stem cells and was predisposed to growing in the extremities, whereas PNET signified all embryonal tumors of the central nervous system, regardless of their sites of origin.^[9]^

As for the treatment of PNET, there some studies have shown that combined modality treatment is a possibly effective treatment for PNET. Surgery is the priority treatment for pathologic confirmation and reducing the tumor volume. RT includes local irradiation and craniospinal irradiation, which depends on the general condition of the patient and the nature of the disease.^[9]^ In recent years, chemotherapy has been showing encouraging results for the treatment of PNET. An induction chemotherapy before tumor excision tends to reduce the tumor size, and also to control systemic disease.^[2]^ But in intrathecal PNET, the blood–brain barrier prevents chemotherapeutic agents from gaining access to the tumor, making it difficult to treat.^[9]^ In our case, no chemotherapy was given after the first operation under the consideration that chemotherapy with multiregimen might also have numerous side effects in children. Hrabálek et al^[10]^ reported a literature review of primary intraspinal PNETs. They concluded that the introduction of chemotherapy and radiation therapy dramatically improved the prognosis of patients with PNET, whereas RT better controlled local disease, and systemic chemotherapy reduced the risk of distant metastasis.^[10]^ In another Chinese study by Tong et al, 13 patients with primary intraspinal PNETs were surgically treated, and during a mean follow-up of 25.5 months, local relapse occurred in 8 patients and distant metastases occurred in 8 patients. The overall 2-year survival rate was 54%, and the 2-year survival rate was 57.1% in patients with adjuvant chemotherapy and 50% in those without chemotherapy. The study concluded gross total resection and adjuvant RT with or without chemotherapy demonstrated a longer survival period (2-year survival rate 86%), and chemotherapy demonstrated a trend for survival benefit in patient with PNET.^[11]^ In a case report and literature review presented by Kiatsoontorn et al, 13 cases were reviewed, where 1 patient received operation followed by RT and 1 patient received operation alone, whereas others received operation with adjuvant chemoradiation. Seven of the 12 previous patients received local adjuvant RT, and 4 patients received craniospinal irradiation. After reviewing literatures, the authors concluded that no evidence was shown that craniospinal axis radiation was more effective than local radiation, and cisplatin-based combination chemotherapy may be the first-line treatment in recurrent or refractory Ewing sarcoma tumors.^[12]^ In a case report with literature review, Sharma et al concluded that maximal safe resection followed by aggressive chemoRT appeared to be the standard treatment. In this study, they also mentioned that because RT could be detrimental to the spinal cord, especially in the pediatric age group, intensity-modulated radiation, and proton beam RT have received attention, which are known to have better dose distribution.^[4]^ In our case, adjuvant RT was given to the primary surgical bed only without craniospinal radiation due to not having obvious sign of leptomeningeal metastasis. As far as we know, craniospinal radiation might have benefit in control of leptomeningeal metastasis, but it might cause some neurologic defects with time. After administrating chemotherapy to our case for tumor recurrence and brain metastasis, the disease has been controlled quite well. Due to limited case numbers of intraspinal PNET, there is no known treatment guideline for such cases. In our case, chemotherapy played an important role in the treatment in intraspinal PNET.

The case we present here demonstrated that PNET is a very aggressive disease, which might have the possibility of recurrence and metastasis. However, because the disease was first diagnosed some time ago, the pathologic image of her first operation was no longer available. The other limitation is that we did not use questionnaires to evaluate her intelligence either before or after whole-brain RT. We evaluated her psychological condition by observation during follow-ups at the outpatient department, which showed no neurological sequelae, and she volunteered to play piano at the concert in the hospital occasionally. Despite the insufficiencies mentioned above, with literatures reviewed above and the case presented here, we concluded that multimodality management indeed demonstrated improved disease control and should be considered in the initial treatment planning in patients with PNET. Chemotherapy and RT are also effective in recurrent PENT with distant metastasis, such as brain metastasis. Further prospective studies might be needed for standard treatment protocol for spinal PNET.

## 4 Conclusions

Multimodality treatments should be considered in initial treatment planning, and salvage chemotherapy can be useful in cases of local recurrence or distant metastasis. Radiation therapy might have a role in disease control.
